# Function and Characteristics of PINK1 in Mitochondria

**DOI:** 10.1155/2013/601587

**Published:** 2013-02-27

**Authors:** Satoru Matsuda, Yasuko Kitagishi, Mayumi Kobayashi

**Affiliations:** Department of Environmental Health Science, Nara Women's University, Kita-Uoya Nishimachi, Nara 630-8506, Japan

## Abstract

Mutations in phosphatase and tensin homologue-induced kinase 1 (PINK1) cause recessively inherited Parkinson's disease, a neurodegenerative disorder linked to mitochondrial dysfunction. Studies support the notion of neuroprotective roles for the PINK1, as it protects cells from damage-mediated mitochondrial dysfunction, oxidative stress, and cell apoptosis. PARL is a mitochondrial resident rhomboid serine protease, and it has been reported to mediate the cleavage of the PINK1. Interestingly, impaired mitophagy, an important autophagic quality control mechanism that clears the cells of damaged mitochondria, may also be an underlying mechanism of disease pathogenesis in patients for Parkinson's disease with the PARL mutations. Functional studies have revealed that PINK1 recruits Parkin to mitochondria to initiate the mitophagy. PINK1 is posttranslationally processed, whose level is definitely regulated in healthy steady state of mitochondria. As a consequence, PINK1 plays a pivotal role in mitochondrial healthy homeostasis.

## 1. Introduction

Mitochondria play an important role in eukaryotic metabolic processes by serving as cellular energy generators of ATP [1], which are critical for cell survival and for correct cellular functions, and they play an important role in mediating apoptosis and in determining their own destruction called mitophagy [2], an important autophagic control mechanism that clears damaged mitochondria. Mitochondria are also recognized to play an important role in neurodegenerative disorders including multiple sclerosis, Alzheimer's, and Parkinson's diseases, which are characterized by progressive and selective loss of neuronal cell populations [3–5]. Midbrain dopaminergic neurons are susceptible to oxidative stress due to the environment of the dopamine biosynthetic pathways and their low mitochondrial reserve compared to other neuronal populations [6]. Molecular genetics has linked mitochondrial dysfunction to the pathogenesis of Parkinson's disease by the discovery of several inherited mutations in gene products that associate with the mitochondrial function.

**Figure 1:**
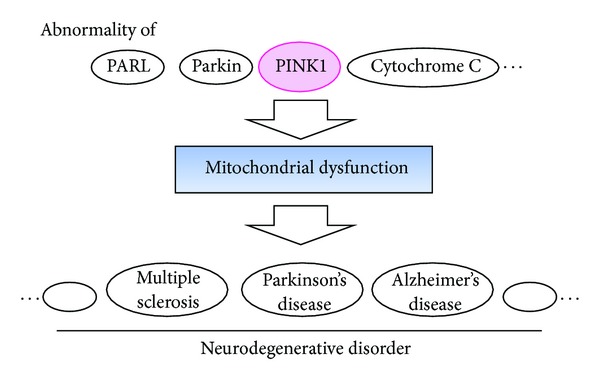
Implication of mitochondrial dysfunction caused by PINK1, Parkin, and so on for neurodegenerative disorders including Parkinson's disease. Abnormality of these molecules may also be a causative factor in the development of mitochondrial dysfunction. There is a relationship between mitochondrial dysfunction and neurodegenerative disorders. Note that some critical molecules have been omitted for clarity.

The PTEN-induced kinase 1 (PINK1) is a mitochondria-targeted serine/threonine kinase, which is linked to autosomal recessive familial Parkinson's disease [7] (Figure 1). In addition to its protective role against mitochondrial dysfunction and apoptosis, PINK1 is also known to regulate Parkinson's disease-related protein Parkin [7]. The PINK1 recruits the E3 ubiquitin ligase Parkin to mitochondria in order to initiate the mitophagy. In addition, presenilin-associated rhomboid-like serine protease (PARL) can affect the proteolytic processing of the PINK1 [8]. Normal PINK1 localization and stability requires catalytic activity of the PARL. Consequently, PARL deficiency impairs Parkin recruitment to mitochondria, suggesting that PINK1 processing and localization is essential in determining its interaction with Parkin [9]. More than 50 mutations of PINK1 have been mapped throughout the kinase and carboxyl-terminal regulatory domains of PINK1 with various effects on protein stability implicating neuroprotective roles [10, 11]. This paper will provide a concise overview on the cellular functions of the mitochondrial kinase PINK1 and the relationship between parkinsonism and mitochondrial dynamics, particular emphasis on a mitochondrial damage response pathway and mitochondrial quality control.

## 2. Expression and Characteristics of PINK1 

**Figure 2:**
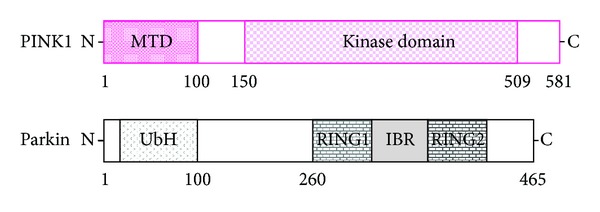
Schematic diagram indicating the domain structures of PINK1 (upper) and Parkin (lower) proteins. The predicted consensual important domain structures for each protein are depicted. MTD: mitochondrial targeting domain, UbH: ubiquitin homology domain, RING1 and RING2: RING finger domain, and IBR: in between RING fingers.

**Figure 3:**
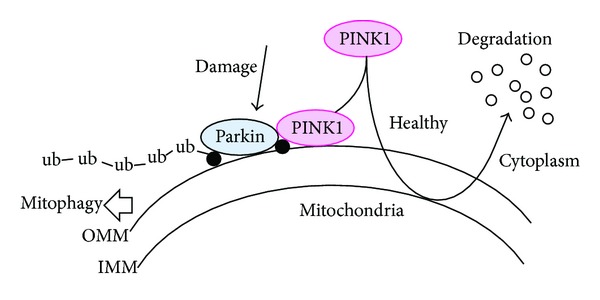
Hypothetical schematic representation of the PINK1 regulatory pathway and Parkin mediated-mitophagy. Under healthy and steady state, PINK1 is degraded within the mitochondria. This may be inhibited by mitochondrial damage, resulting in PINK1 and Parkin accumulation in the outer membrane of mitochondria. Parkin is presumed to ubiquitinate unidentified substrate (black circle), resulting in the induction of mitophagy. Note that some critical pathways have been omitted for clarity. OMM: outer mitochondrial membrane; IMM: inner mitochondrial membrane.

Mutations in PINK1 are the most common cause of recessive familial Parkinsonism [10, 11]. The *PINK1 (phosphatas-e and tensin-homolog- (PTEN-)induced kinase 1)* gene consists of eight exons, encoding a 581-amino acid protein with a predicted molecular mass of 62.8 kilodaltons. Defects in the *PTEN*, which is a tumor suppressor, have been found in cancers arising in a variety of human tissues. *PINK1* mRNA is expressed ubiquitously, but high expression levels are found in the heart, skeletal muscle, testes, and brain [12]. In the brain, higher expression is neuronal in the substantia nigra, hippocampus, and cerebellar Purkinje cells [13]. The PINK1 protein has a central domain with homology to serine/threonine kinases, exhibiting an auto-phosphorylation activity *in vitro *[14]. An amino-terminal mitochondrial-targeting signal domain is sufficient for mitochondrial introduction of PINK1 (Figure 2) [15]. The protein can be found on the outer and inner mitochondrial membrane (Figure 3) [16, 17]. The PINK1 can be processed into at least two shorter forms, which are distributed in both mitochondrial and cytosolic compartments. Physiological PINK1 substrates are localized in the outer mitochondrial membrane or possibly in the cytosol near the mitochondrial surface. The cytoplasmic PINK1 is degraded by proteasome [18]. Adding to the variety of survival functions of PINK1, it has been shown to phosphorylate the mitochondrial heat shock protein 75 kDa (TRAP1), increasing neuronal survival against oxidative stress or heat shock by preventing the release of cytochrome c [19]. The mitochondrial serine protease HtrA2 has been identified to be regulated by PINK1 [20]. Targeted deletion of the HtrA2 causes mitochondrial dysfunction leading to a neurodegenerative disorder with parkinsonian features in mice [20]. The TRAP1 may be a direct substrate for PINK1, which localize primarily in the mitochondrial matrix and at extramitochondrial sites. Whether HtrA2 is a direct PINK1 substrate is somewhat unclear. It is possible that differences in cell viability resulting from PINK1 inactivation may affect HtrA2 through the other kinase such as p38 SAPK [21]. HtrA2 is released from the inter membrane space of mitochondria during apoptosis to the cytosol [22, 23]. PINK1 may also interact with Beclin1, a key proautophagic protein implicated in the pathogenesis of Alzheimer's and Huntington's diseases [24]. Full-length PINK1 interacts with Beclin1 [25], which enhances starvation-induced autophagy. The PARL is a mitochondrial resident rhomboid serine protease and has been reported to mediate the cleavage of PINK1 in mitochondria, which may mediate differential cleavage of PINK1 and phosphoglycerate mutase 5 (PGAM5) depending on the health status of mitochondria [26].

## 3. PINK1 Function Involved in Mitochondrial Health Status 

PINK1 silencing may result in mitochondrial respiratory dysfunction, since PINK1 knockout mice exhibit impaired mitochondrial respiration and decreased activity of oxidative phosphorylation [27]. In addition, the impaired mitochondrial respiration can be exacerbated by exposure of the mitochondria to heat shock [27]. While knockdown studies of endogenous PINK1 indicate a key role for the PINK1 in maintaining the mitochondrial functioning networks, the protective activities of PINK1 depend on its mitochondrial localization. Loss of PINK1 leads to severe alterations in mitochondrial homeostasis as evidenced by increased mitochondrial reactive oxygen species (ROS) inducing a robust increase in mitochondrial mitophagy [28]. Stable PINK1 silencing may have an indirect role in the mitophagy activation. As proteins with iron sulfur clusters, one of the most ubiquitous redox centers, are sensitive to oxidative stress, prolonged ROS exposure may cause mitochondrial dysfunction [29, 30]. PINK1 has been shown to protect against cell death induced by proteasome inhibition and oxidative damage [31, 32]. Thus, PINK1 has a pivotal role in the mitochondrial quality control via the mitochondrial stabilization, phosphorylation of chaperones, and regulation of the mitophagy. However, imbalanced induction of mitophagic recycling can contribute to neuronal atrophy, neurite degeneration, and neuronal cell death [33]. Excessive rates of mitophagy may prove harmful results [33, 34]. Overexpression of wild-type PINK1 in neuronal cells stabilizes respiring mitochondrial networks through maintaining mitochondrial membrane potential and suppression of the mitophagy [35]. Probably, high levels of cytoplasmic PINK1 may substitute for endogenous protein by phosphorylating substrates at the mitochondrial surface or in the cytoplasm near the mitochondrial surface. In healthy mitochondria, PINK1 is rapidly degraded in a process involving both mitochondrial proteases and the proteasome. The mitochondrial protease PARL can affect the proteolytic processing of PINK1 and normal PINK1 localization, and the stability requires the PARL catalytic activity [17, 36]. The PARL may also mediate differential cleavage of PINK1 depending on the health status of mitochondria. PARL deficiency impairs Parkin recruitment to mitochondria, suggesting that PINK1 processing and localization are important in determining its interaction with the Parkin.

With severe mitochondrial damage, PINK1 facilitates aggregation of depolarized mitochondria through interactions with Parkin protein [37]. In addition, overexpression of full-length PINK1 is required for mitochondrial Parkin recruitment for the mitochondrial aggregation. Besides, transient overexpression of Parkin further augments mitochondrial mitophagy in PINK1 deficient neuronal cells, resulting in cytoprotection and/or restoration of interconnected mitochondrial networks [38]. Many lines evidences indicate interactions of PINK1 with Parkin in promoting mitochondrial health homeostasis [38, 39]. The Parkin can be phosphorylated by PINK1 in its RING finger domain during *in vitro* kinase reactions, which may promote translocation of the Parkin to mitochondria [40]. Furthermore, the phosphorylated Parkin has been reported to facilitate the selective clearance of depolarized mitochondria via mitophagy [41, 42]. Under conditions of PINK1 diminishment or deficiency, it compromises the mitochondrial quality control. Failure of this mitochondrial quality control eventually contributes to cell death. In healthy mitochondria, by the way, PINK1 is rapidly degraded in a process involving both mitochondrial proteases and the proteasome as mentioned above. Loss of either PINK1 or Parkin leads to fragmentation of mitochondria [43, 44]. On the other hand, mitochondrial Parkin promotes the mitophagic degradation of dysfunctional mitochondria [25, 45]. The mitophagic response observed in PINK1 silencing cells could be associated with increased Parkin levels, as endogenous Parkin protein expression is increased in some PINK1 deficient cells [46]. Thus, PINK1 and Parkin could complexly participate in a common mitochondrial protective signaling pathway.

## 4. Abnormal PINK1 Involved in Neurodegenerative Disease

Intramembrane proteolysis is a conserved mechanism that regulates various cellular processes. The PARL cleaves human PINK1 within its conserved membrane anchor [47], suggesting implication in neurodegenerative disease. Mature PINK1 is then free to be released into the cytosol or into the mitochondrial intermembrane space. Upon depolarization of the mitochondrial membrane potential, the import of PINK1 and PARL-catalyzed processing is blocked, leading to accumulation of the PINK1 precursor [47]. Targeting of this precursor to the outer mitochondrial membrane has been shown to trigger the mitophagy (Figure 3) [48]. The PARL-catalyzed removal of the PINK1 signal sequence in the import pathway may act as a cellular checkpoint for mitochondrial integrity. Interestingly, Parkinson's disease-causing mutations decrease the processing of PINK1 by PARL [49]. Decreased processing of Pink1 may be an implication for the pathogenesis. When mitochondrial import is compromised by depolarization, PINK1 accumulates on the mitochondrial surface, where it recruits the Parkinson's disease-linked Parkin from the cytosol, which in turn mediates the mitophagic destruction of mitochondria (Figure 3) [48, 49]. The importance of PINK1 in mechanisms underlying neurodegeneration is reflected by the neuroprotective properties of the Parkin in counteracting oxidative stress and improvement of mitochondrial function. The involvement of Parkin and PINK1 in mitochondrial dysfunction, oxidative injury, and impaired functioning of the ubiquitin-proteasome system has been investigated in light of Parkinson's disease pathogenesis [48, 49].

A protein kinase microtubule-affinity regulating kinase 2 (MARK2) also plays key roles in several cell processes underlying neurodegenerative diseases, such as Alzheimer's disease, by phosphorylating tau and detaching it from microtubules [50]. MARK2 phosphorylates and activates the PINK1 [51]. Thr-313 is the primary phosphorylation site, a residue mutated to a nonphosphorylatable form in a frequent variant of Parkinson's disease [51]. Mutation of the Thr-313 in PINK1 shows toxic effects with abnormal mitochondrial distribution in neurons. Both MARK2 and PINK1 have been found to colocalize with mitochondria and regulate their transport. So, MARK2 may be an upstream regulator of PINK1, and it regulates the mitochondrial trafficking in neuronal cells. The MARK2-PINK1 cascade provides new insights into the regulation of mitochondrial trafficking in neurons and neurodegeneration in Parkinson's disease. The high temperature requirement A2 (HtrA2) is indirectly phosphorylated and interacts with PINK1 in relation to a signaling pathway [52]. The PINK1-dependent phosphorylation of the HtrA2 enhances its protease activity leading to enhanced survival against oxidative stress [52, 53]. The HtrA2 is also phosphorylated on activation of the p38 SAPK pathway, occurring in a PINK1-dependent manner [52]. Point mutations in the HtrA2 are a susceptibility factor for Parkinson's disease. However, it has been shown in Drosophila that the HtrA2 is not essential for all the protective functions of PINK1 [54, 55]. Another mitochondrial protease rhomboid-7 has been implicated in posttranslational regulation of both PINK1 and HtrA2 [56, 57]. These protein signaling axes might provide a link between neurodegenerative processes in Alzheimer's and Parkinson's diseases.

## 5. Perspective

Mitochondrial protein phosphorylation is involved in cell stress-induced programmed cell death such as apoptosis, which also contributes to the regulation of mitochondrial dynamics and mitophagy. Those are significant to maintain mitochondrial quality and ensure cellular homeostasis. PINK1 may function in the first line of mitochondrial quality control, monitoring respiratory chain function [58] and triggering the localized degradation of damaged mitochondrial proteins. In addition, diminishment of PINK1 would have deleterious consequences on mitochondrial function [59]. The PINK1 is a mitochondrial kinase that promotes cell survival, particularly under conditions of oxidative stress. Whether PINK1 levels are enhanced or reduced, strategies to promote selective mitophagy and mitochondrial biogenesis may prove to be effective for multiple forms of neurodegenerative disease. Although the precise physiological substrate of PINK1 is not fully resolved, it is clear that the kinase activity is important in playing roles for many aspects of mitochondrial function [60, 61]. The involvement of PINK1 and Parkin in the mitochondrial dysfunction has now been intensively investigated in Parkinson's disease pathogenesis [62]. These pathological mechanisms are not restricted to the Parkinson's disease, but they might be common characters of various neurodegenerative and neuroinflammatory disorders. It is therefore conceivable that PINK1 and Parkin are also linked to the pathogenesis of other neurological diseases including Alzheimer's disease. The mechanisms by which wild-type PINK1 and Parkin promote interconnected mitochondrial networks may involve different steps in mitochondrial quality control. For example, severe mitochondrial injury may require organelle-level responses including Parkin-facilitated mitochondrial mitophagy. Enhancing pathways that promote mitophagy might also delay age-related diseases by promoting a healthy pool of viable mitochondria in neuronal cells and sustaining energy demands. Future experimental work would be needed to understand the precise mitochondria protective roles of PINK1.

## Abbreviations

HtrA2: High temperature requirement protein A2
MARK2: Microtubule affinity-regulating kinase 2
PARL: Presenilin-associated rhomboid-like serine protease
PINK1: PTEN-induced kinase1, phosphatase and tensin homologue-induced kinase 1
PGAM5: Phosphoglycerate mutase 5
PTEN: Phosphatase and tensin homolog
ROS: Reactive oxygen species
SAPK: Stress-activated protein kinase
TRAP1: Tumor necrosis factor receptor-associated protein 1.
